# Microbial Consortiums of Hydrogenotrophic Methanogenic Mixed Cultures in Lab-Scale *Ex-Situ* Biogas Upgrading Systems under Different Conditions of Temperature, pH and CO

**DOI:** 10.3390/microorganisms8050772

**Published:** 2020-05-21

**Authors:** Jun Xu, Fan Bu, Wenzhe Zhu, Gang Luo, Li Xie

**Affiliations:** 1 The Yangtze River Water Environment Key Laboratory of the Ministry of Education, College of Environmental Science and Engineering, Tongji University, Shanghai 200092, China; 1830587@tongji.edu.cn (J.X.); bufan@tongji.edu.cn (F.B.); wzz@tongji.edu.cn (W.Z.); 2 Shanghai Key Laboratory of Atmospheric Particle Pollution and Prevention (LAP3), Department of Environmental Science and Engineering, Fudan University, Shanghai 200092, China; gangl@fudan.edu.cn; 3 Shanghai Institute of Pollution Control and Ecological Security, Shanghai 200092, China

**Keywords:** biogas upgrading, hydrogenotrophic methanogens, microbial community, temperature, pH, CO

## Abstract

In this study, hydrogenotrophic methanogenic mixed cultures taken from 13 lab-scale *ex-situ* biogas upgrading systems under different temperature (20–70 °C), pH (6.0–8.5), and CO (0–10%, *v*/*v*) variables were systematically investigated. High-throughput 16S rRNA gene sequencing was used to identify the microbial consortia, and statistical analyses were conducted to reveal the microbial diversity, the core functional microbes, and their correlative relationships with tested variables. Overall, bacterial community was more complex than the archaea community in all mixed cultures. Hydrogenotrophic methanogens *Methanothermobacter*, *Methanobacterium,* and *Methanomassiliicoccus*, and putative syntrophic acetate-oxidizing bacterium *Coprothermobacter* and *Caldanaerobacter* were found to predominate, but the core functional microbes varied under different conditions. Multivariable sensitivity analysis indicated that temperature (*p* < 0.01) was the crucial variable to determine the microbial consortium structures in hydrogenotrophic methanogenic mixed cultures. pH (0.01 < *p* < 0.05) significantly interfered with the relative abundance of dominant archaea. Although CO did not affect community (*p* > 0.1), some potential CO-utilizing syntrophic metabolisms might be enhanced. Understanding of microbial consortia in the hydrogenotrophic methanogenic mixed cultures related to environmental variables was a great advance to reveal the microbial ecology in microbial biogas upgrading process.

## 1. Introduction

Biogas upgrading via an external H_2_ supply to promote CO_2_ biomethanation has attracted considerable attention [1]. Hydrogenotrophic methanogens play an important role in the efficient biogas upgrading process and can capture CO_2_ by combining it with H_2_ to produce CH_4_ [2,3]. It was reported that the methane content in biogas could be increased up to 89–96% by hydrogenotrophic methanogens in various *in-situ* and *ex-situ* CO_2_ biomethanation processes [4,5,6,7,8]. Microbial analysis conducted in various biogas upgrading configurations revealed that the *Methanobacterium*, *Methanothermobacter*, *Methanoculleus*, and *Methanomicrobium* species were dominant in the hydrogenotrophic methanogenic consortium [3,5,9,10,11,12]. The utilization of hydrogenotrophic methanogenic mixed cultures enriched by H_2_ were found to be more efficient in CO_2_ biomethanation, giving larger CH_4_ yields compared to the utilization of pure cultures [13], for example, of *Methanothermobacter thermautotrophicus* [14]. In addition, the practical application of mixed adapted cultures is preferable as compared with pure cultures [13], due to its advantages of the greater robustness of the microbial community, flexible process control, and increased cost-effectiveness [15,16,17].

The inherent sensitivity of anaerobic microbes determines the higher request of environmental conditions [18]. Hydrogenotrophic archaeal species and their abundance in mixed cultures are influenced by fermentation temperature, pH, and other variables. In a previous study, it was reported that different archaeal genera within the order Methanobacteriales were observed in the mixed cultures at 37 and 55 °C, respectively [4]. Another study indicated that the archaeal genus *Methanoculleus* belonging to the order Methanomicrobiales was dominant in the systems, but the relative abundance decreased from 36 to 24% as the temperature increased from 37 to 55 °C [7]. Moreover, further increasing the temperature to 65 or 70 °C was more advantageous for specific hydrogenotrophic methanogenic activity, with higher strain performance. The dominant archaea genus shifted to *Methanobacterium* from *Methanothermobacter* [19]. Regarding the variable of pH, pH 8.5 representing alkaline conditions was found to be the threshold value for optimal *in-situ* H_2_/CO_2_ biomethanation at both 35 and 55 °C [7]; however, the community shift in the *ex-situ* system has seldom been described. With the exception of external variables, the incoming gas composition also highly influenced the microbial community in mixed cultures. For instance, carbon monoxide (CO) is a common component in alternative H_2_ abundant gas (such as syngas and coke oven gas) for biogas upgrading. It was demonstrated that the archaeal genus *Methanoculleus* was positively selected when CO was introduced into the *in-situ* biogas upgrading reactor operating at 37 °C, with the relative abundance increasing from 0.4 to 45.9% compared to non-CO systems dominated by the order Methanosarcinales [20]. Our previous research exhibited a higher abundance of the dominant genus *Methanothermobacter* in a CO-containing *ex-situ* biogas upgrading reactor (40.6 and 99.0% at 55 and 70 °C, respectively), and the potential syntrophic relationship between the bacterial genus *Coprothermobacter* and archaeal genus *Methanothermobacter* was enhanced at both temperatures [21].

All these studies undoubtedly confirm that the interdependent microorganisms in systems respond sensitively to environmental variables. In recent years, the majority of studies regarding hydrogenotrophic methanogen distribution and structure in the microbiological biogas upgrading process have been conducted either in the *in-situ* or *ex-situ* bioreactors, with different inocula, complex substrates, and various nutrient media. Specific microbes contributing to H_2_/CO_2_ biomethanation under certain conditions were respectively identified. However, systematic investigations of the community assembly and its correlation with different multivariable co-influences have seldom been reported. Understanding the microbial community shift in hydrogenotrophic methanogenic mixed cultures related to different variables, e.g., temperature, pH, and CO conditions, would represent a great advance and help to reveal the microbial ecology in the microbial biogas upgrading process [22].

On the basis of the above rationale, the aim of this study was to evaluate the co-influences of temperature, pH, and CO variables on the syntrophic microbial community and the core hydrogenotrophic methanogens in lab-scale *ex-situ* biogas upgrading reactors. The mixed cultures, collected from different semi-continuous reactors after long-term and stable operation, were conducted using 16S rRNA gene high-throughput sequencing technology. After quality control, the alpha diversity and microbial community of mixed cultures were assessed. In addition, the sensitivities of the hydrogenotrophic methanogenesis-related microorganisms to different conditions were characterized. Furthermore, redundancy analysis (RDA) and canonical correlation analysis (CCA) were used to determine the correlative relationship between archaea, bacterial community, and the tested variables.

## 2. Materials and Methods

### 2.1. Sampling of Hydrogenotrophic Methanogenic Mixed Cultures

The hydrogenotrophic methanogenic mixed cultures were collected from different semi-continuous *ex-situ* biogas upgrading reactors after long-term and stable acclimation under the different variables tested in our lab. Each bioreactor was fed with CO_2_ as the sole carbon source or additionally provided with a certain amount of CO (0%, 5%, and 10%, *v*/*v*) under different temperature (20, 30, 55, 65, or 70 °C) and pH (6.0, 7.5, or 8.5) conditions. The operational parameters of each bioreactor for sludge sampling are described in Table 1. The specific procedures regarding the acclimation of hydrogenotrophic methanogenic mixed cultures under different operational conditions are described in Supplementary File S1, among which the details about nutrient medium (Table S1) and bioreactors (Table S2) are included. All the sludge samples were stored at −80 °C prior to DNA extraction.

### 2.2. High-Throughput 16S rRNA GENE Sequencing

#### 2.2.1. DNA Extraction

In this study, total community genomic DNA was extracted using an E.Z.N.A.^®^ Mag-Bind Soil DNA Kit (Omega Bio-tek, Norcross, GA, USA) according to the manufacturer’s instructions. The DNA concentration was measured using a Qubit 2.0 fluorimeter (Life Technologies, Waltham, MA, USA), and the quality of the DNA was checked using an Agilent 2100 Bioanalyzer (Agilent Technologies, Santa Clara,, CA, USA) to ensure that adequate amounts of high-quality genomic DNA were extracted.

#### 2.2.2. Polymerase Chain Reaction

Polymerase chain reaction (PCR) was conducted immediately after DNA extraction. The 16S rRNA V3–V4 gene was amplified using KAPA HiFi Hot Start ready mix (TaKaRa Bio Inc., Shiga, Japan). Archaeal DNA was amplified using the forward primer 340F (CCCTAYGGGGYGCASCAG) and the reverse primer 1000R (GGCCATGCACYWCYTCTC) to generate the first-round amplicon and the primers 349F (GYGCASCAGKCGMGAAW) and 806R (GGACTACVSGGGTATCTAAT) to generate the second-round amplicon [23] with the reactions set up as follows: 2 μL of microbial DNA (10 ng/μL), 1 μL each of the forward and reverse primers (10 μM), and 15 μL of 2 × KAPA HiFi Hot Start Ready Mix (total volume of 30 μL). The plate was sealed and PCR was performed using an Applied Biosystems 9700 instrument (Thermo Fisher Scientific, MA, USA) and the following program: 1 cycle of denaturation at 95 °C for 3 min; 5 cycles of denaturation at 95 °C for 30 s, annealing at 45 °C for 30 s, and elongation at 72 °C for 30 s; followed by 20 cycles of denaturation at 95 °C for 30 s, annealing at 55 °C for 30 s, and elongation at 72 °C for 30 s; with a final extension at 72 °C for 5 min. Bacterial DNA was amplified using the PCR primers forward 341F (CCTACGGGNGGCWGCAG) and reverse 805R (GACTACHVGGGTATCTAATCC), with the reaction set up as described for the archaeal PCR [23]. The PCR products were assessed via electrophoresis in 1% (*w*/*v*) agarose gels in TBE buffer (Tris, boric acid, and EDTA) stained with ethidium bromide (EB) and visualized under UV light.

#### 2.2.3. DNA Purification and Quantitative Mixing

AMPure XP beads (Beckman Coulter, USA) were used to remove the free primers and primer dimer species from the amplicon products. A library was constructed using the universal Illumina adaptor and index. Depending on the coverage needs, all libraries were pooled for one run. Amplicons from each reaction mixture were pooled in equimolar ratios based on their concentration. The reaction products were sequenced using the Illumina MiSeq system (Illumina, San Diego, CA, USA) according to the manufacturer’s instructions. After sequencing, data were collected as follows: (1) Two short Illumina readings were assembled by PEAR (v0.9.6) software according to the overlap, and fastq files were processed to generate individual fasta and qual files, which could then be analyzed by standard methods; (2) Sequences containing ambiguous bases as well as any longer than 480 base pairs (bp) were dislodged and those with a maximum homopolymer length of 6 bp were allowed [24]. In addition, sequences shorter than 200 bp were removed; (3) All identical sequences were merged into one; (4) Sequences were aligned according to a customized reference database; (5) The intactness of the index and the adaptor was checked, and all sequence information corresponding to the index and adaptor sequences was then removed; (6) Noise was removed using the Pre.cluster tool. Chimeras were detected using Chimera UCHIME. All the software was in the Mothur package [25].

### 2.3. Statistical Data Analysis

All the sequences were grouped into operational taxonomic units (OTUs) using a program from the Mothur package [26]. Effective sequences after processing were submitted to the Ribosomal Database Project (RDP) Classifier to confirm the archaeal and bacterial sequences.

#### 2.3.1. Alpha Diversity Analysis

The microbial diversity of each single sample was estimated by alpha diversity analysis. In this study, species richness and diversity statistics, including the Coverage, Chao1, ACE, Simpson, and Shannon indices, were calculated according to the OTU results. Then, all the effective sequences without primers were submitted for downstream analysis [27]. 

#### 2.3.2. Microbial Community Analysis

The microbial community was illustrated using a heatmap, presenting the composition of microorganisms and their relative abundances. Thereafter, the core functional archaea and bacteria in the hydrogenotrophic methanogenic mixed culture were analyzed using network diagrams calculated using the Kamada–Kawai algorithm with R programming language and the Vegan package.

#### 2.3.3. Correlative Analysis Between Microbial Community and Environmental Variables

Correlation coefficients of single microorganisms to each environmental variable were evaluated by Pearson’s correlation algorithm. Eventually, the multivariable sensitivity of the overall hydrogenotrophic methanogenic mixed cultures was revealed by redundancy analysis (RDA) [28] and canonical correlation analysis (CCA) [29] with R programming language and the Vegan package. The selection of RDA or CCA was determined by gradient lengths of the detrended correspondence analysis. If the value of its first axis was lower than 3.5, CCA was used, otherwise the RDA was more appropriate.

## 3. Results and Discussion

### 3.1. Overview of Community Diversity under Various Temperature, pH, and CO Conditions

Microbial DNA sequences were generated through Miseq from the hydrogenotrophic methanogenic mixed cultures. After quality control, a total of 527,952 archaeal sequences and 560,780 bacterial sequences were obtained. These sequences were subsequently clustered into OTUs at 97% sequence similarity. With the purpose of the assessing the internal complexity of microbial community, index of Coverage, Chao, ACE, Shannon and Simpson indices were investigated. A coverage estimator > 0.999 indicates that the majority of bacterial and archaeal OTUs were captured, guaranteeing the reliability of the sequencing results (Table 2). As shown in Table 2, the number of OTUs, as well as values for the richness and evenness indices of the bacterial communities, were significantly higher than those of the archaea community, demonstrating that the bacterial community was more complex than the archaea community in the hydrogenotrophic methanogenic mixed culture. 

In the archaeal community, values for both Chao and ACE indices were lower than 16 in the 55B, 70B, and 70N_10 mixed cultures, indicating that the corresponding conditions presented higher selectivity towards archaea diversity. By contrast, for the 20N, 30N, 55N_5, 70N, and 70A samples, the conditions were relatively beneficial for the richness of the archaea community, with observed Chao and ACE index values greater than 24. Further comparisons of the Shannon and Simpson indices for different mixed cultures revealed that there was regularity behind the variety of indices. The evenness deteriorated with increased temperature in the archaeal community, represented by lower Shannon but higher Simpson values. Non-neutral pH negatively influenced the archaeal community, and the values for the Simpson index, for example, followed the order 55A > 55B > 55N and 70A > 70B > 70N, suggesting that the majority of archaea survived under neutral conditions (pH of 7.0). It was reported that the decreased archaeal diversity under alkaline conditions could have contributed to the greater energy consumption required to sustain the intracellular pH balance [30]. In this study, acidic conditions were found to severely inhibit archaeal community diversity. This might result from the presence of acid-sensitive archaea in the hydrogenotrophic methanogenic mixed cultures. 

As for the bacterial community, the corresponding conditions for the 20N and 30N samples were more favorable than for the others, with both Chao and ACE indices higher than 290 (Table 2). Overall, the richness of the bacterial community decreased with the gradual increase in temperature, with lower values for Chao and ACE indices obtained at 55 and 70 °C, which a similar trend for the archaeal community. Notably, the growth of bacterial communities remained relatively unstimulated at non-neutral pH. It is interesting that bacterial diversity in the alkaline hydrogenotrophic methanogenic mixed culture was enhanced under extreme-thermophilic conditions (for example, a Chao index of 110.11 for 70N and 223.84 for 70B). This result might due to the fact that the dominant bacteria consortium in the mixed culture was susceptible to alkaline conditions, such that the relative abundances of other bacteria were increased as a result of their higher alkaline tolerance. Further evidence is presented in the following sections.

With the exception of temperature and pH variables, the microbial characteristics related to CO introduction in terms of OTUs as well as Coverage, richness, and evenness indices exhibited irregular fluctuations under various temperature and pH conditions. Therefore, the microbial community composition and structure in different hydrogenotrophic methanogenic mixed cultures should be further investigated.

### 3.2. Microbial Community Structure of Hydrogenotrophic Methanogenic Mixed Cultures

**Figure 1:**
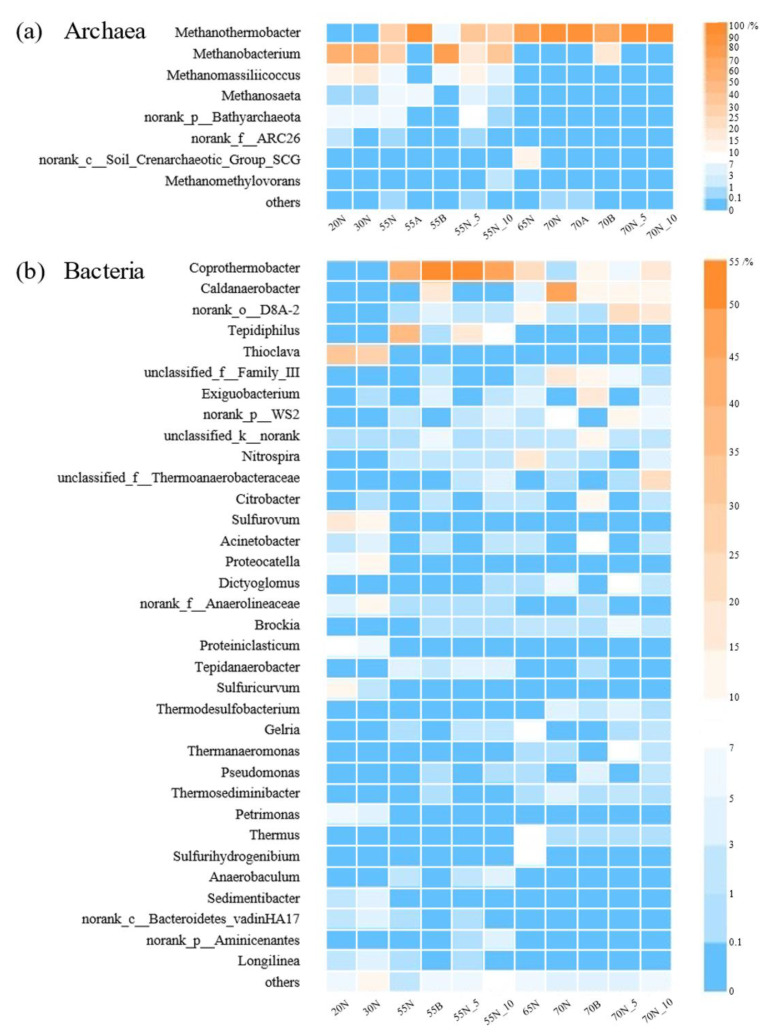
Heatmaps of the microbial communities at the genus level: (**a**) archaea; (**b**) bacteria. The unclassified/no-rank microbial genera in the mixed cultures are marked with their upstream taxonomic name; for example, the no-rank archaea at the genus level within phylum Bathyarchaeota is described as norank p Bathyarchaeota.

Taxonomic classification at the genus level was systematically examined to verify the structure of microbial communities. Overall, 8 archaeal genera and 34 bacterial genera (relative abundance > 3%) were identified and are illustrated in Figure 1 (the relative abundance of all detected microbial genera in the mixed cultures are provided in Tables S3 and S4). Figure 1a shows the structures of the archaeal communities at the genus level in a heatmap. As can be seen, hydrogenotrophic methanogens dominated in all samples with a narrow distribution, mainly including *Methanothermobacter*, *Methanobacterium*, and *Methanomassiliicoccus*. The above three genera accounted for 79.2–99.8% of archaeal abundance in total. By contrast, the typical acetoclastic archaea *Methanosaeta* showed inferior growth in all hydrogenotrophic methanogenic mixed cultures with a relative abundance of less than 9.5%. The other genera were marginally detected in partial samples due to their competitive disadvantage.

As illustrated from Figure 1a, the sequences related to the genera *Methanobacterium* (67.6% and 65.4% in the 20N and 30N samples, respectively) and *Methanomassiliicoccus* (18.6% and 20.6% in the 20N and 30N samples, respectively) showed a relatively high abundance in the above-mixed cultures. With the temperature increasing to 55, 65, and 70 °C, the genus *Methanothermobacter* demonstrated dominance over *Methanobacterium* and *Methanomassiliicoccus* with a relative abundance of 38.0–97.7%, due to its favorable growth at higher temperatures [31]. This finding was in close agreement with a previous study wherein *Methanothermobacter* dominated the archaeal communities in *ex-situ* methanation systems at 55 and 65 °C [8]. Interestingly, pH was a significant factor of microbial abundance perturbation that contributed to a 22.1–30.1% decrease in *Methanothermobacter*, but a 23.31–50.8% increase in *Methanobacterium* under alkaline conditions. A pH of 8.5 seemed to greatly alleviate the negative effects of the extreme-thermophilic condition for *Methanobacterium*. The higher alkaline adaptability of the genus *Methanobacterium* was consistent with the results of a site remediation study wherein *Methanobacterium* sp. dominated in a lime kiln filtrate [32]. Additionally, it is noteworthy that the introduction of CO may not severely interfere with the dominant archaeal genera, as it has been reported that *Methanothermobacter* spp. are capable of converting CO to CH_4_ [33,34]. The relative abundance of *Methanothermobacter* in the samples where CO was introduced remained at 35.1–41.3% and 99.0–99.8% at 50 and 70 °C, respectively.

In terms of bacterial relative abundance, significant dominance was observed with two bacterial phyla, namely Firmicutes and Proteobacteria (61.2–88.0%). As illustrated in Figure 1b, the genera *Thioclava* and *Sulfurovum* were the most abundant at lower temperatures (a total of 47.8 and 36.9% in the 20N and 30N samples, respectively), followed by *Proteocatella* (6.3–14.1%), *Sulfuricurvum* (1.8–12.1%), norank family Anaerolineaceae (3.2–11.0%), *Proteiniclasticum* (5.7–9.1%), *Sedimentibacter* (2.9–4.2%) and *Longilinea* (1.8–3.5%). By comparison, a remarkable spatial difference was demonstrated whereby the genus *Coprothermobacter* dominated in thermophilic mixed cultures with a relative abundance of 42.8 to 59.0%, and *Caldanaerobacter* abundance was prominently increased, accounting for 10.9–49.2% in extreme-thermophilic mixed cultures. These observations are all in good accordance with their reported optimal growth temperatures [35,36]. Notably, *Coprothermobacter* was reported as a typical syntrophic bacterial genus that contributes to the degradation of small-molecule organic compounds to promote hydrogen production, subsequently providing sufficient raw materials for hydrogenotrophic methanogens [37,38]. Moreover, the genus *Coprothermobacter* was also a potential syntrophic acetate oxidizer interacting with methanogens at 55 °C, as previous reported [39]. Hence, it might be involved in converting acetate to H_2_ and CO_2_, thus providing hydrogenotrophic methanogens with a slight amount of carbon for further biomethanation. Additionally, *Caldanaerobacter*, which dominated at 70 °C, was also a potential syntrophic acetate oxidation bacterium (SAOB) that established an intimate syntrophic association with extreme-thermophilic methanogens in the mixed cultures [40]. It is interesting that four abundant bacterial genera *Tepidiphilus*, *Exiguobacterium*, norank order D8A-2, and *Tepidanaerobacter*, where were subdominant in the thermophilic and extreme-thermophilic samples, were also reported as potential SAOBs [41,42,43,44]. These syntrophic genera, together with *Coprothermobacter* and *Caldanaerobacter*, accounted for the majority of bacterial abundance, ranging from 39.9 to 86.0%, implying the pathway of syntrophic acetate oxidation coupled with hydrogenotrophic methanogenesis (SAO-HM) existed in the hydrogenotrophic methanogenic mixed culture. Moreover, the total proportion of SAOBs that dominated in the samples followed the sequence of 55N (83.1%) > 55B (79.5%) > 70N (50.8%) > 70B (41.7%), indicating the pathway of SAO-HM might be weakened at 70 °C, especially under alkaline conditions. Besides, CO introduction seemed to interfere with the bacterial proportions by augmenting *Coprothermobacter* abundance by 5.5–16.3% but significantly decreasing *Caldanaerobacter* abundance by 34.8–36.2%. Coincidentally, in a previous investigation on simultaneous sewage sludge treatments and CO biomethanation, an increased *Coprothermobacter* abundance was also observed in the liquid phase after CO addition [25]. It is noteworthy that although some species belonging to the genus *Caldanaerobacter* have been reported to utilize CO as a substrate [45], they may suffer a comparative competitive disadvantage in hydrogenotrophic methanogenic mixed cultures. Overall, the bacterial communities in hydrogenotrophic methanogenic mixed cultures were abundant with SAOBs, of course, as well as a small amount of various indispensable bacteria.

### 3.3. Correlative Relationship Between Microorganisms and Temperature, pH, and CO

The correlative relationship between the environmental variables (temperature, pH, and CO) and the relative abundance of microbial genera in the hydrogenotrophic methanogenic mixed cultures were examined using Pearson correlation analysis. Archaeal and bacterial genera that were significantly correlated with at least one of the environmental variables are summarized in Table 3.

The overall archaeal community dissimilarities were primarily attributed to the temperature and pH variables, to which they were negatively correlated. Specifically, *Methanobacterium* (r = −0.893, *p* < 0.01), *Methanomassiliicoccus* (r = −0.932, *p* < 0.01), norank phylum Bathyarchaeota (r = −0.823, *p* < 0.01), norank phylum ARC26 (r = −0.812, *p* < 0.01), and norank family Terrestrial Miscellaneous Gp TMEG (r = −0.603, 0.05 < *p* < 0.1) demonstrated a negative relationship with temperature, while *Methanothermobacter* (r = −0.732, 0.01 < *p* < 0.05), *Methanosarcina* (r = −0.584, 0.05 < *p* < 0.1), *Methanobrevibacter* (r = −0.725, 0.01 < *p* < 0.05), *Methanoculleus* (r = −0.824, *p* < 0.01), *Methanospirillum* (r = −0.650, *p* < 0.1), *Methanosphaera* (r = −0.568, *p* < 0.1), and norank family Thermoplasmatales Incertae Sedis (r = −0.826, *p* < 0.01) exhibited a significant negative correlative relationship with pH. The megatrends of archaeal abundance decline related to increasing temperature and pH variables were highly consistent to the alpha diversity results in Section 3.1, indicating high temperature and pH were unfavorable to the majority of methanogens in hydrogenotrophic methanogenic mixed cultures. However, a significant positive correlation coefficient was observed between the genus *Methanothermobacter* and temperature (r = −0.560, 0.05 < *p* < 0.01), explaining the predominance of *Methanothermobacter* over all the archaeal genera in the extreme-thermophilic mixed cultures. In terms of CO introduction, in general, the archaeal abundance was insignificantly influenced. Although the genus *Methanomethylovorans* showed a positive significant correlation to increasing CO concentration (r = 0.603, *p* < 0.1), the maximum relative abundance of 3.5% was negligible compared to the dominant *Methanothermobacter* or *Methanobacterium*.

Dynamic changes in the bacterial community as a result of temperature, pH, and CO variables exhibited a higher complexity than for archaea. As observed in Table 3, a total of 19 bacterial genera were significantly influenced by temperature, among which, 13 genera were positively enriched but 6 genera were negatively inhibited. By contrast, higher pH and CO introduction seemed to be favorable for bacterial communities, with 6 and 3 positively correlative genera, respectively. Potential syntrophic metabolism was implied by the enhancement of abundance for members of the unclassified family Thermoanaerobacteraceae in response to CO introduction (r = 0.753, 0.0 5 < *p* < 0.01). The family Thermoanaerobacteraceae has been previously reported to be capable of utilizing CO. Alves et al. [46] suggested that the genus *Thermoanaerobacter* belonging to family Thermoanaerobacteraceae showed high resistance to CO-containing environments. This contributed to the conversion of CO into direct materials for hydrogenotrophic methanogenesis or acetotrophic methanogenesis. For instance, *Thermoanaerobacter thermohydrosulfuricus* subsp. *carboxydovorans* (stain TDL) was able to convert CO/H_2_O to CO_2_/H_2_ [45], and *Thermoanaerobacter kivui* was capable of capturing CO as a single-electron source for CH_3_COOH/H_2_ formation and coupling with energy conservation for oxidation [47]. In this study, genus unclassified family Thermoanaerobacteraceae accounted for 4.0% and 21.3% of total bacterial abundance in 10% CO-containing samples at 55 and 70 °C, respectively, while it was fairly indetectable in non-CO mixed cultures. These results strongly suggest the possibility of syntrophic CO-utilization associated with methanogens in the hydrogenotrophic methanogen mixed cultures. Additionally, CO acclimation at 70 °C increased the relative abundance of *Dictyoglomus* (from 6.4 to 8.3%), *Brockia* (from 1.1 to 6.3%) and *Thermanaeromonas* (from 0.2 to 8.1%), which were identified as sulfur-reducing bacteria [48,49,50]. It was likely that the supplied CO promoted electron transfer for the sulfur reduction. However, the relative abundance of the three abovementioned sulfur-reducing bacteria reduced to 1.8%, 2.8%, and 1.2% after 10% CO exposure, indicating that a higher CO concentration might not be favorable for sulfur-reducing syntrophic associations. Conversely, the abundance of the genus *Coprothermobacter* increased both at 55 °C (from 41.3 to 50.3%) and 70 °C (from 0.3 to 15.3%), implying that the SAO-HM pathway established by *Coprothermobacter* was enhanced by CO [21].

**Figure 2:**
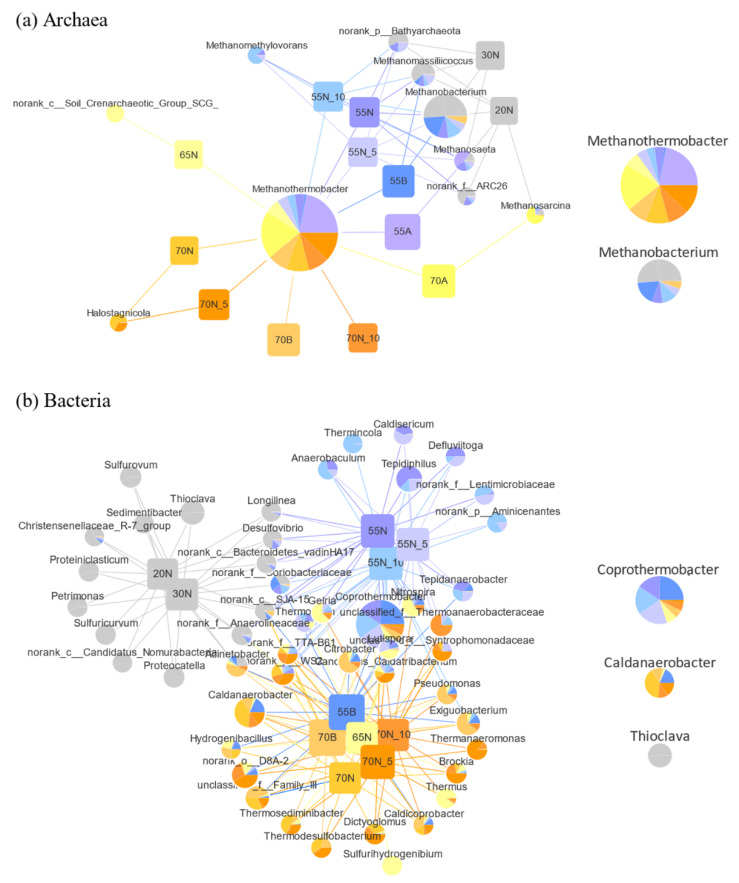
Profile clustering Cytoscape network visualizing microbial genera and their associations with mixed cultures: (**a**) archaea (relative abundance > 0.1%); (**b**) bacteria (relative abundance > 1%).

Furthermore, a profile network analysis was constructed to identify the core functional microorganisms in the hydrogenotrophic methanogenic mixed cultures. After qualified clustering, 10 effective archaeal nodes (relative abundance > 0.1%) and 49 effective bacterial nodes (relative abundance > 1%) related to hydrogenotrophic methanogenic mixed cultures were identified and are illustrated in Figure 2. The genus *Methanothermobacter* was observed as the core functional archaeal microbe (degree of 11) in the majority of hydrogenotrophic methanogenic mixed cultures. This result is highly consistent with the previous observation that the genus *Methanothermobacter* is responsible for the majority of biological hydrogen methanation [51]. However, it was replaced by the sub-core functional genus *Methanobacterium* (degree of 6) in the 20N and 30N samples due to an unfavorable environmental temperature of below 40 °C [52]. Regarding bacterial microbes, *Coprothermobacter* was identified as the core functional bacterial microbe (degree of 9) despite it being undetected in 20N and 30N. It is noteworthy that the sub-core functional bacterial genus *Caldanaerobacter* (degree of 9) was also inexistent at 20 and 30 °C. Conversely, it was dominated by the genus *Thioclava* (degree of 2). Actually, more than half of the bacterial nodes connected to 20N and 30N were unidentified under thermophilic or extreme-thermophilic conditions. Overall, this demonstrates that the growth and presence of the core functional bacterial microbes are very much determined by environmental temperature variables.

### 3.4. The Multivariable Sensitivity of Hydrogenotrophic Methanogenic Mixed Cultures

**Figure 3:**
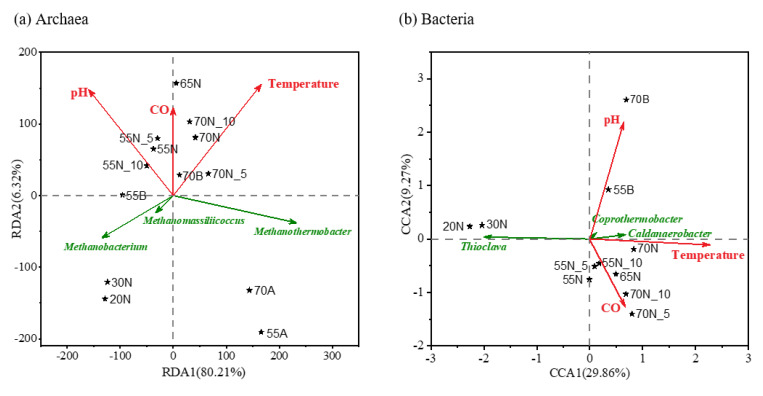
Redundancy analysis (RDA) and canonical correlation analysis (CCA) of microbial communities related to environmental variables: (**a**) archaeal community; (**b**) bacterial community. Nodes: hydrogenotrophic methanogenic mixed culture samples; red arrows: environmental variables; green arrows: functional microbes.

RDA and CCA were further performed to assess the contributions of environmental variables (temperature, pH, and CO) to variances in the archaeal and bacterial communities. As illustrated in Figure 3a,b, the length of the red arrows indicates the extent to which the microbial community characteristics were influenced by the environmental variables. For archaeal community, the relative influence of the environmental variables followed the sequence of temperature (r^2^ = 0.8658, *p* < 0.01), pH (r^2^ = 0.6335, 0.01 < *p* < 0.05), and CO (r^2^ = 0.1546, *p* > 0.1). This suggests that temperature and pH significantly determine the characteristics of the archaeal community in hydrogenotrophic methanogenic mixed cultures. Interestingly, a similar multivariable sensitivity was observed in the bacterial community where the temperature (r^2^ = 0.9524, *p* < 0.01) and pH (r^2^ = 0.8143, 0.01< *p* < 0.05) significantly influenced the bacterial structure in the hydrogenotrophic methanogenic mixed cultures. Although the insignificant correlation of CO introduction (r^2^ = 0.3106, *p* > 0.1) was maintained, the increasing r^2^ (from 0.1546 to 0.3106) implies an enhancement of the potential syntrophic associations in CO utilization.

Additionally, the co-influences of multivariable on hydrogenotrophic methanogenic mixed cultures are implied by the angle between the environmental variable arrows. Specifically, angle < 90° represents a positive relationship, while angle > 90° indicates a negative relationship. In Figure 3a, the angles between the three variables demonstrate that a positive relationship existed between temperature, pH, and CO co-influence on archaeal community. This suggests that the introduction of any two variables contributed, to a certain extent, to promoting the changing tendency of the overall archaeal community caused by the third variable. By contrast, a negative co-influence was observed between pH and CO in the bacterial community when angle > 90° (Figure 3b). This implies a mutually restricted relationship between the influence of the pH and CO variables on the overall bacterial community. After comparison, the bacterial community in hydrogenotrophic methanogenic mixed cultures showed higher multivariable sensitivity than the archaeal community regarding temperature, pH, and CO.

It is noteworthy that the angle between the environmental variables and functional microbes confirmed the correlative relationship observed in the above section. For instance, an angle between the pH variable and the core functional archaeal genus *Methanothermobacter* of >90° demonstrated the negative influence of pH on the relative abundance of *Methanothermobacter*, which was highly consistent with the Pearson correlation coefficients (r = −0.732, 0.01 < *p* < 0.05) in Section 3.3. Similarly, the positive influence of the temperature variable on the genus *Methanothermobacter* was confirmed by the angle between the temperature arrow and *Methanothermobacter* (<90°), which also closely coincided with the correlation analysis (r = 0.560, 0.05 < *p* < 0.1). For the same reason, the dependence of the sub-core functional archaeal genera on variables of temperature, pH, and CO was clearly illustrated and exhibited identical results as before. The same relationship results as were mentioned in Section 3.3 were observed in the bacterial community (Figure 3b).

## 4. Conclusions

Hydrogenotrophic methanogenic consortia, with hydrogenotrophic methanogens *Methanothermobacter*, *Methanobacterium* and *Methanomassiliicoccus*, and putative SAOB *Coprothermobacter* and *Caldanaerobacter* were eventually constructed in lab-scale *ex-situ* biogas upgrading systems. Temperature and pH significantly affected the microbial community. Dominant archaea and bacteria shifted from *Methanobacterium* to *Methanothermobacter*, and *Coprothermobacter* to *Caldanaerobacter*, respectively, with increasing temperature from 20 to 70 °C. Notably, *Methanothermobacter* deteriorated but *Methanobacterium* was augmented under alkaline conditions at high temperature. While CO introduction did not change the dominance of *Methanothermobacter* in mixed cultures, but some potential syntrophic association was probably enhanced. Tested environmental variables affected the archaea structure followed the sequence of temperature, pH, and CO.

## Figures and Tables

**Table 1:** Information regarding the operational parameters and CH_4_ production performance of each acclimation bioreactor.

Bioreactor	20N	30N	55N	55A	55B	55N_5	55N_10	65N	70N	70A	70B	70N_5	70N_10
Total volume (mL)	300	300	300	620	620	620	620	300	300	620	620	620	620
Working volume (mL)	100	100	100	300	300	300	300	100	100	300	300	300	300
Initial VS (g/L)	20	20	20	30	30	30	30	20	20	30	30	30	30
Temperature (°C)	20	30	55	55	55	55	55	65	70	70	70	70	70
pH	7.5 ± 0.2	7.5 ± 0.2	7.5 ± 0.2	6.0 ± 0.2	8.5 ± 0.2	7.5 ± 0.2	7.5 ± 0.2	7.5 ± 0.2	7.5 ± 0.2	6.0 ± 0.2	8.5 ± 0.2	7.5 ± 0.2	7.5 ± 0.2
Daily CO_2_ addition (Nml)	37.3	37.3	45.8	74.5	74.5	74.5	74.5	45.8	45.8	74.5	74.5	74.5	74.5
H_2_:CO_2_(*v*/*v*) ^a^	5:1	5:1	5:1 *	5:1	5:1	-	-	5:1 *	5:1 *	5:1	5:1	-	-
H_2_:CO_2:_CO(*v*/*v*)	-	-	-	-	-	80:16:5	80:16:11	-	-	-	-	80:16:5	80:16:11
Carbon-loading rate (×10^−4^ mol/g VS) ^b^	8.33	8.33	10.22	3.70	3.70	4.85	6.24	10.22	10.22	3.70	3.70	4.85	6.24
Speed (rpm)	120 ± 5	120 ± 5	120 ± 5	120 ± 5	120 ± 5	120 ± 5	120 ± 5	120 ± 5	120 ± 5	120 ± 5	120 ± 5	120 ± 5	120 ± 5
HRT(days)	10	10	10	10	10	10	10	10	10	10	10	10	10
Average daily CH_4_ production (NmL)	30	30	43.3 ± 3.0	76.5 ± 7.5	77.6 ± 6.8	94.4 ± 3.8	102 ± 2.2	46.1 ± 2.3	46.9 ± 1.6	63.9 ± 11.4	73.9 ± 8.7	89.5 ± 4.6	99.4 ± 2.8

^a^ The H_2_/CO_2_(*v*/*v*) ratio in the 55N, 65N, and 70N (noted with “*”) bioreactors was changed to 4:1 under the prerequisite that enough hydrogen was present for complete CO_2_ conversion, while those for other bioreactors remained unchanged despite sufficient hydrogen being available. ^b^ The carbon-loading rate was described as CO and/or CO_2_ addition per unit volatile solid (VS). in the bioreactors. The influence of carbon-loading rate was neglected due to the fact that adequate sludge existed for complete conversion from carbon oxide to methane. It was confirmed with the redundancy analysis/canonical correlation analysis (RDA/CCA) analysis (*p* > 0.1).

**Table 2:** Sequencing results and alpha diversity indices of the archaeal and bacterial communities.

Sample	Archaea						Bacteria					
	OTUs	Coverage	Richness		Evenness		OTUs	Coverage	Richness		Evenness	
			Chao	ACE	Shannon	Simpson			Chao	ACE	Shannon	Simpson
20N	26	1.0000	26.33	26.91	1.60	0.32	269	0.9992	290.25	291.15	3.03	0.11
30N	25	0.9999	26.00	32.05	1.79	0.24	282	0.9995	295.80	293.80	3.28	0.09
55N	22	1.0000	22.00	22.00	1.62	0.27	223	0.9992	254.14	253.03	1.79	0.31
55A ^a^	16	1.0000	16.00	16.34	0.33	0.83	N/A	N/A	N/A	N/A	N/A	N/A
55B	11	1.0000	11.00	11.00	0.74	0.66	206	0.9992	251.04	248.11	2.09	0.31
55N_5	25	0.9999	28.00	32.20	1.65	0.26	245	0.9992	261.35	271.12	2.01	0.35
55N_10	16	0.9999	17.00	19.36	1.35	0.35	209	0.9991	265.89	252.44	2.37	0.27
65N	13	0.9998	19.00	20.97	0.51	0.71	167	0.9995	177.22	183.28	2.90	0.10
70N	21	0.9998	26.00	25.14	0.68	0.60	89	0.9995	110.11	107.21	2.24	0.19
70A ^a^	24	1.0000	24.00	24.26	0.17	0.94	N/A	N/A	N/A	N/A	N/A	N/A
70B	9	1.0000	9.00	9.00	0.64	0.62	186	0.9993	223.84	221.85	2.67	0.10
70N_5	14	0.9999	17.00	16.15	0.42	0.78	71	0.9998	84.00	83.98	2.66	0.10
70N_10	12	0.9999	13.00	16.66	0.41	0.76	88	0.9995	107.09	113.10	2.59	0.12

^a^ The bacterial DNA sequences in 55A and 70A samples were not obtained.

**Table 3:** Correlation coefficients between abundant microbial genera and environmental variables.

Microbial Genera	Temperature	pH	CO
**Archaeal**			
*Methanothermobacter*	0.560 *	−0.732 **	−0.077
*Methanobacterium*	−0.893 ***	0.369	−0.202
*Methanomassiliicoccus*	−0.932 ***	0.138	−0.164
*Methanosaeta*	−0.246	−0.530	−0.161
Norank p Bathyarchaeota	−0.823 ***	0.067	−0.168
Norank f ARC26	−0.812 ***	0.032	−0.186
*Methanosarcina*	0.129	−0.584 *	−0.185
*Methanomethylovorans*	−0.051	0.029	0.603 *
Norank f Terrestrial Miscellaneous Gp TMEG	−0.603 *	0.055	−0.131
*Methanobrevibacter*	0.058	−0.725 **	−0.239
*Methanoculleus*	0.228	−0.824 ***	−0.056
*Methanospirillum*	0.083	−0.650 *	−0.315
Unclassified k norank	−0.007	−0.702 **	−0.302
*Methanosphaera*	−0.036	−0.568 *	−0.179
Norank f Thermoplasmatales Incertae Sedis	0.171	−0.836 ***	−0.263
**Bacterial**			
*Caldanaerobacter*	0.537 *	0.266	−0.167
*Thioclava*	−0.911 ***	−0.222	−0.329
Unclassified f Family_III	0.525 *	0.455	−0.277
*Exiguobacterium*	0.346	0.775 **	−0.148
Norank p WS2	0.572 *	−0.438	0.391
unclassified k norank	0.306	0.888 ***	−0.269
Unclassified f Thermoanaerobacteraceae	0.286	−0.21	0.753 **
*Citrobacter*	0.344	0.779 **	−0.152
*Sulfurovum*	−0.916 ***	−0.219	−0.324
*Acinetobacter*	0.023	0.686 *	−0.269
*Proteocatella*	−0.762 **	−0.201	−0.297
Norank f Anaerolineaceae	−0.704 *	−0.172	−0.284
*Proteiniclasticum*	−0.915 ***	−0.218	−0.322
*Sulfuricurvum*	−0.792 **	−0.174	−0.257
*Thermodesulfobacterium*	0.534 *	0.053	−0.088
*Pseudomonas*	0.34	0.761 **	−0.144
*Caldicoprobacter*	0.603 **	0.627 *	−0.228
*Petrimonas*	−0.917 ***	−0.219	−0.325
Norank f TTA-B61	0.589 *	−0.435	0.606 *
*Anaerobaculum*	−0.029	−0.244	0.497
*Sedimentibacter*	−0.842 **	−0.214	−0.318
Norank c Bacteroidetes vadinHA17	−0.82 **	−0.207	−0.321
Norank p Aminicenantes	−0.022	−0.179	0.624 *
*Longilinea*	−0.792 **	−0.207	−0.314
Norank c Candidatus_Nomurabacteria	−0.916 ***	−0.22	−0.326
*Christensenellaceae* R-7 group	−0.846 **	−0.173	−0.331
Norank c SJA-15	−0.584	−0.039	−0.297
Norank f Lentimicrobiaceae	−0.04	−0.237	0.568
*Hydrogenibacillus*	0.486	0.568	−0.517
*Thermincola*	−0.018	−0.154	0.592
*Desulfovibrio*	−0.936 ***	−0.248	−0.357

Significance: *** *p* < 0.01; ** 0.01 < *p* < 0.05; * 0.05 < *p* < 0.1.

## Supplementary Materials

Supplementary materials can be found at https://www.mdpi.com/2076-2607/8/5/772/s1.
