# Supplementary material for: Microbial Consortiums of Hydrogenotrophic Methanogenic Mixed Cultures in Lab-Scale Ex-Situ Biogas Upgrading Systems under Different Conditions of Temperature, pH and CO
Source: Microorganisms. 2020 May 21;8(5):772. doi: 10.3390/microorganisms8050772 (PMC7285331; doi:10.3390/microorganisms8050772)
Supplement: Supplementary file 1 [file microorganisms-08-00772-s001.zip › Manuscript-supplementary/Supplementary File S1. Acclimations of hydrogenotrophic methanogenic mixed cultures..docx]

**Supplementary File S1: Acclimations of hydrogenotrophic methanogenic mixed cultures.**

The origin inoculum for the acclimation experiment was obtained from a mesophilic up-flow anaerobic sludge blanket (UASB) reactor treating wastewater from a local paper mill in Shanghai, China. The collected granular sludge was stored at −20 °C, and was slowly thawed at ambient temperature then washed with distilled water before being used as the acclimation inoculum. Basal anaerobic (BA) medium was used as nutrient medium in the acclimation experiments as per Angelidaki and Sanders [1]. The stock solutions for BA medium were prepared as described in Table S1. The following volumes of stock solutions were mixed and diluted to a total volume of 1 L with distilled water: A) 10 mL; B) 2 mL; C) 1 mL; D) 1 mL; and E) 1 mL. The mixture was flushed with 100% N_2_ before being used. Cysteine hydrochloride (0.5 g) was added and the medium was dispensed to serum vials, and autoclaved if necessary.

Table S1. Stock solutions of basal anaerobic (BA) medium.

| Component | Reagent |
| --- | --- |
| A | NH_4_Cl, 100 g/L; NaCl, 10 g/L; MgCl_2_·6H_2_O, 10 g/L; CaCl_2_·2H_2_O, 5 g/L; |
| B | K_2_HPO_4_·3H_2_O, 200 g/L; |
| C | Resazurin, 0.5 g/L |
| D | FeCl_2_·4H_2_O, 2 g/L; H_3_BO_3_, 0.05 g/L; ZnCl_2_, 0.05 g/L; CuCl_2_·2H_2_O, 0.038 g/L; MnCl_2_ 4H_2_O, 0.05 g/L; (NH_4_)_6_Mo_7_O_24_·4H_2_O, 0.05 g/L; AlCl_3_, 0.05 g/L; CoCl_2_·6H_2_O, 0.05 g/L; NiCl_2_·6H_2_O, 0.092 g/L; EDTA, 0.5 g/L; concentrated HCl, 1 ml; Na_2_SeO_3_·5H_2_O,0.1 g/L; |
| E | Biotin, 2.0 mg/L; Folic acid, 2.0 mg/L; Pyridoxal HCl, 10.0 mg/L; Thiamine HCl, 5.0 mg/L; Riboflavin, 5.0 mg/L; Nicotnic acid, 5.0 mg/L; Ca-D-Pantotheinate, 5.0 mg/L; Cyanocobalamin, 0.1 mg/L; P-aminobenzoic acid, 5.0 mg/L; Thioctic acid, 5.0 mg/L; |

A series of pressure resistant high-borosilicate bottles were used as bioreactors for the acclimation of hydrogenotrophic methanogens under different temperature, pH, and CO conditions. The information regarding the distinct conditions for each bioreactor is detailed in Table S2. The temperature was controlled using shaking water baths set at a speed of 120 ± 5 rpm, the pH was adjusted using HCl or NaOH, and extra CO was supplied according to the indicated concentration.

Table S2. Information regarding the operational parameters and CH4 production performance of each acclimation bioreactor

| Bioreactor | 20N | 30N | 55N | 55A | 55B | 55N_5 | 55N_10 | 65N | 70N | 70A | 70B | 70N_5 | 70N_10 |
| --- | --- | --- | --- | --- | --- | --- | --- | --- | --- | --- | --- | --- | --- |
| Total volume (mL) | 300 | 300 | 300 | 620 | 620 | 620 | 620 | 300 | 300 | 620 | 620 | 620 | 620 |
| Working volume (mL) | 100 | 100 | 100 | 300 | 300 | 300 | 300 | 100 | 100 | 300 | 300 | 300 | 300 |
| Initial VS (g/L) | 20 | 20 | 20 | 30 | 30 | 30 | 30 | 20 | 20 | 30 | 30 | 30 | 30 |
| Temperature (℃) | 20 | 30 | 55 | 55 | 55 | 55 | 55 | 65 | 70 | 70 | 70 | 70 | 70 |
| pH | 7.5±0.2 | 7.5±0.2 | 7.5±0.2 | 6.0±0.2 | 8.5±0.2 | 7.5±0.2 | 7.5±0.2 | 7.5±0.2 | 7.5±0.2 | 6.0±0.2 | 8.5±0.2 | 7.5±0.2 | 7.5±0.2 |
| Daily CO_2_ addition (Nml) | 37.3 | 37.3 | 45.8 | 74.5 | 74.5 | 74.5 | 74.5 | 45.8 | 45.8 | 74.5 | 74.5 | 74.5 | 74.5 |
| H_2_:CO_2_(v/v) ^a^ | 5:1 | 5:1 | 5:1* | 5:1 | 5:1 | - | - | 5:1* | 5:1* | 5:1 | 5:1 | - | - |
| H_2_:CO_2:_CO(v/v) | - | - | - | - | - | 80:16:5 | 80:16:11 | - | - | - | - | 80:16:5 | 80:16:11 |
| Carbon-loading rate ^b^  (×10^-4^ mol /g) | 8.33 | 8.33 | 10.22 | 3.70 | 3.70 | 4.85 | 6.24 | 10.22 | 10.22 | 3.70 | 3.70 | 4.85 | 6.24 |
| Speed (rpm) | 120±5 | 120±5 | 120±5 | 120±5 | 120±5 | 120±5 | 120±5 | 120±5 | 120±5 | 120±5 | 120±5 | 120±5 | 120±5 |
| HRT(days) | 10 | 10 | 10 | 10 | 10 | 10 | 10 | 10 | 10 | 10 | 10 | 10 | 10 |
| Average daily CH_4_ production (NmL） | 30 | 30 | 43.3±3.0 | 76.5±7.5 | 77.6 ± 6.8 | 94.4 ± 3.8 | 102±2.2 | 46.1±2.3 | 46.9±1.6 | 63.9±11.4 | 73.9 ±8.7 | 89.5 ± 4.6 | 99.4 ± 2.8 |

^a^ The H_2_/CO_2_(*v*/*v*) ratio in the 55N, 65N, and 70N bioreactors was changed to 4:1 under the prerequisite that enough hydrogen was present for complete CO_2_ conversion, while those for other bioreactors remained unchanged despite sufficient hydrogen being available.

^b^ The carbon-loading rate was described as CO and/or CO_2_ addition per unit volatile solid VS. in the bioreactors. The influence of carbon-loading rate was neglected due to the fact that adequate sludge existed for complete conversion from carbon oxide to methane. It was confirmed with the redundancy analysis/canonical correlation analysis (RDA/CCA) analysis (*p* > 0.1)

Initially, the mixture of the processed inoculum and BA medium was respectively added into each bioreactor. All the reactors were flushed for several minutes with high-purity H_2_ (purity > 99.99%) and sealed, and then the H_2_/CO_2_ ratio of headspace gas was adjusted by injecting pure CO_2_ (purity > 99.99%). Extra CO (purity > 99.99%) was supplied for four CO-containing reactors according to specified concentration. The acclimation experiment was conducted for several months. Both liquid and gas were replaced regularly (details about the liquid phase and gas phase are not shown, and further acclimation data can be found in our previous work [2-3]). Specifically, the BA medium along with an equal volume of sampling liquid was used to replenish the bioreactors, and the pH of the acclimation culture was readjusted to the corresponding value. In addition, the headspace was re-injected with H_2_ and CO_2_ (or CO_2_ / CO) by repeating the above procedure. The success of microbial acclimation was determined through long-term stable performance, e.g., the efficiency of carbon resource utilization, VFA concentration in liquid, biogas productivity, methane production rate (data not shown).

[1] Irini Angelidaki, Wendy Sanders. Assessment of the anaerobic biodegradability of macropollutants[J]. Re/Views in Environmental ence & Bio/Technology, 2004, 3(2):117-129.

[2] Dong, N. S.; Bu, F.; Zhou, Q.; Khanal, S. K.; Xie, L., Performance and microbial community of hydrogenotrophic methanogenesis under thermophilic and extreme-thermophilic conditions. *Bioresource Technol* 2018, *266*, 454-462.

[3] Bu, F.; Dong, N. S.; Khanal, S. K.; Xie, L.; Zhou, Q., Effects of CO on hydrogenotrophic methanogenesis under thermophilic and extreme-thermophilic conditions: Microbial community and biomethanation pathways. *Bioresource Technol* 2018, *266*, 364-373.
