# Supplementary material for: Microbial Consortiums of Hydrogenotrophic Methanogenic Mixed Cultures in Lab-Scale Ex-Situ Biogas Upgrading Systems under Different Conditions of Temperature, pH and CO
Source: Microorganisms. 2020 May 21;8(5):772. doi: 10.3390/microorganisms8050772 (PMC7285331; doi:10.3390/microorganisms8050772)
Supplement: Supplementary file 1 [file microorganisms-08-00772-s001.zip › Manuscript-supplementary/Supplementary Table S3. Relative abundance (%) of all detected archaeal genera in the mixed cultures..docx]

**Supplementary Table S3. Relative abundance (%) of all detected archaeal genera in the mixed cultures.**

| Archaeal genus | 20N | 30N | 55N | 55A | 55B | 55N_5 | 55N_10 | 65N | 70N | 70A | 70B | 70N_5 | 70N_10 |
| --- | --- | --- | --- | --- | --- | --- | --- | --- | --- | --- | --- | --- | --- |
| g__*Methanothermobacter* | - | - | 38.01 | 90.65 | 7.87 | 41.29 | 35.08 | 82.47 | 97.71 | 97.42 | 75.65 | 98.98 | 99.84 |
| g__*Methanobacterium* | 67.64 | 65.46 | 32.91 | 0 | 83.76 | 23.50 | 48.70 | 0.08 | 0.04 | 0.19 | 23.32 | 0.04 | 0.01 |
| g__*Methanomassiliicoccus* | 18.61 | 20.59 | 8.30 | 0.01 | 7.61 | 16.72 | 6.60 | 0 | 0.09 | 0 | 0.84 | 0.01 | 0.01 |
| g__*Methanosaeta* | 1.54 | 2.83 | 9.53 | 9.08 | 0.06 | 5.20 | 3.85 | 0.02 | 0.04 | 0.13 | 0.08 | 0.08 | 0.05 |
| g__norank_p__Bathyarchaeota | 7.00 | 9.55 | 7.61 | 0 | 0.62 | 10.70 | 1.19 | 0.16 | 0.07 | 0.02 | 0.10 | 0.01 | - |
| g__norank_f__ARC26 | 4.61 | 0.89 | 2.17 | 0.02 | 0.02 | 1.02 | 0.71 | 0.02 | 0.01 | 0.03 | - | 0 | - |
| g__norank_c__Soil_Crenarchaeotic_Group_SCG_ | - | - | - | - | - | - | - | 17.20 | - | 0.01 | - | - | - |
| g__*Methanosarcina* | 0.33 | 0.15 | 0.15 | 0.05 | 0.03 | 0.08 | 0.31 | - | 0.11 | 2.04 | 0.01 | 0.01 | 0.01 |
| g__*Methanomethylovorans* | 0.01 | 0 | 0.44 | - | - | 0.46 | 3.47 | - | - | 0 | - | - | - |
| g__*Halostagnicola* | 0.01 | - | 0.01 | - | - | 0.03 | - | - | 1.82 | - | - | 0.75 | - |
| g__norank_f__Terrestrial_Miscellaneous_Gp_TMEG_ | 0.21 | 0.47 | 0.62 | - | 0.01 | 0.75 | 0 | - | - | - | - | - | 0.01 |
| g__*Methanolinea* | 0.01 | 0.02 | 0.21 | - | - | 0.22 | 0.09 | 0.04 | - | - | - | - | 0.01 |
| g__*Methanobrevibacter* | - | - | - | 0.10 | - | - | - | - | 0.02 | 0.04 | - | 0 | 0 |
| g__*Methanoculleus* | - | - | - | 0.03 | - | 0 | - | 0 | - | 0.03 | - | 0.01 | 0.04 |
| g__*Methanospirillum* | 0.01 | 0 | - | 0.01 | - | - | - | 0 | 0.02 | 0.04 | - | - | - |
| g__*Natronorubrum* | - | - | - | - | - | - | - | - | 0.05 | - | - | 0.05 | - |
| g__unclassified_k__norank | - | 0.02 | - | 0.01 | - | - | - | - | - | 0.03 | - | - | - |
| g__norank_c__WCHA1-57 | 0.01 | 0 | 0.02 | - | - | 0.01 | 0 | - | - | - | - | - | 0.03 |
| g__*Halalkalicoccus* | - | - | - | - | - | - | - | - | 0.02 | - | - | 0.04 | - |
| g__*Methanosphaera* | - | - | - | 0.02 | - | - | - | - | - | - | - | - | - |
| g__*Methanoregula* | 0 | - | 0.02 | - | - | 0.01 | - | - | - | 0.01 | - | - | - |
| g__norank_f__Thermoplasmatales_Incertae_Sedis | - | - | - | 0.01 | - | - | - | - | - | 0.01 | - | - | - |
| g__Rice_Cluster_I | 0 | 0.01 | 0.01 | - | - | 0 | - | - | - | - | - | - | - |
| g__*Natronococcus* | - | - | - | - | - | - | - | - | 0.01 | - | - | - | - |

Under relative abundance, “-” indicates that the genus was not detected while “0” indicates the genus was detected but at extremely low levels.
