# Supplementary material for: Microbial Consortiums of Hydrogenotrophic Methanogenic Mixed Cultures in Lab-Scale Ex-Situ Biogas Upgrading Systems under Different Conditions of Temperature, pH and CO
Source: Microorganisms. 2020 May 21;8(5):772. doi: 10.3390/microorganisms8050772 (PMC7285331; doi:10.3390/microorganisms8050772)
Supplement: Supplementary file 1 [file microorganisms-08-00772-s001.zip › Manuscript-supplementary/Supplementary Table S4. Relative abundance (%) of all detected bacterial genera in the mixed cultures..docx]

**Supplementary Table S4. Relative abundance (%) of all detected bacterial genera in the mixed cultures.**

| Bacterial genus | 20N | 30N | 55N | 55B | 55N_5 | 55N_10 | 65N | 70N | 70B | 70N_5 | 70N_10 |
| --- | --- | --- | --- | --- | --- | --- | --- | --- | --- | --- | --- |
| g__*Coprothermobacter* | - | - | 42.77 | 56.12 | 59.03 | 53.33 | 22.55 | 0.26 | 11.81 | 5.72 | 15.42 |
| g__*Caldanaerobacter* | - | - | - | 15.85 | - | 0.05 | 3.08 | 49.24 | 10.89 | 14.46 | 13.05 |
| g__norank_o__D8A-2 | - | - | 0.83 | 4.57 | 1.22 | 1.11 | 10.70 | 1.73 | 0.36 | 24.55 | 19.41 |
| g__*Tepidiphilus* | - | - | 38.65 | 0.11 | 15.60 | 8.61 | 0 | 0.01 | 0 | 0.03 | 0 |
| g__*Thioclava* | 32.34 | 26.86 | - | - | - | - | - | - | 0 | - | - |
| g__unclassified_f__Family_III | - | - | - | 1.10 | - | 0.02 | 1.22 | 15.99 | 13.80 | 6.08 | 0.86 |
| g__*Exiguobacterium* | 0.03 | 0.08 | - | 4.29 | - | 2.37 | 3.57 | 0 | 19.29 | - | 4.37 |
| g__norank_p__WS2 | - | - | 2.92 | 0.04 | 2.83 | 3.47 | 2.54 | 8.56 | 0.04 | 10.13 | 5.07 |
| g__unclassified_k__norank | 0.70 | 0.55 | 0.76 | 5.47 | 0.76 | 0.96 | 1.12 | 0.94 | 13.04 | 1.80 | 0.92 |
| g__*Nitrospira* | - | - | 1.35 | 2.50 | 1.91 | 2.20 | 18.54 | 0.95 | 0.13 | 0.04 | 3.42 |
| g__unclassified_f__Thermoanaerobacteraceae | - | - | - | - | 2.87 | 4.23 | - | 0.13 | 0 | 0.66 | 21.55 |
| g__*Citrobacter* | 0.04 | 0.08 | 0 | 2.78 | 0 | 1.60 | 2.41 | 0 | 12.03 | 0 | 2.61 |
| g__*Sulfurovum* | 15.45 | 10.05 | 0 | 0 | 0 | 0 | - | - | - | - | - |
| g__*Acinetobacter* | 1.79 | 4.30 | - | 1.56 | - | 0.97 | 1.28 | 0 | 7.37 | 0 | 1.60 |
| g__*Proteocatella* | 6.31 | 14.08 | - | - | - | - | - | - | - | - | - |
| g__*Dictyoglomus* | - | - | 0 | 0.03 | 0 | 0.24 | 0.73 | 6.42 | 0.05 | 8.36 | 1.81 |
| g__norank_f__Anaerolineaceae | 3.22 | 11.02 | 0.60 | 0.38 | 0.56 | 0.27 | 0 | 0 | 0.09 | - | 0 |
| g__*Brockia* | - | - | 0.01 | 0.17 | 0.20 | 0.42 | 1.74 | 1.11 | 0.43 | 6.36 | 2.79 |
| g__*Proteiniclasticum* | 9.09 | 5.65 | - | - | - | 0 | - | - | - | - | - |
| g__*Tepidanaerobacter* | - | - | 3.75 | 1.21 | 2.93 | 3.14 | 0.03 | 0 | 0.10 | - | 0 |
| g__*Sulfuricurvum* | 12.13 | 1.77 | - | - | - | - | - | - | - | - | - |
| g__*Thermodesulfobacterium* | - | - | - | - | - | 0.02 | 0 | 4.81 | 1.84 | 3.86 | 0.27 |
| g__*Gelria* | 0 | 0.03 | 0.41 | 0 | 1.02 | 1.01 | 7.61 | 0.07 | 0 | 0.29 | 1.03 |
| g__*Thermanaeromonas* | - | - | - | 0 | - | 0 | 0.08 | 0.24 | 0 | 8.13 | 1.22 |
| g__*Pseudomonas* | 0.05 | 0.03 | 0 | 0.89 | 0 | 0.59 | 0.81 | 0 | 4.45 | 0 | 0.96 |
| g__*Caldicoprobacter* | - | 0.02 | 0.19 | 0.74 | 0.13 | 0.17 | 0.59 | 1.86 | 2.41 | 1.46 | 0.47 |
| g__*Thermosediminibacter* | - | - | - | 0.13 | - | 0 | 0.83 | 4.91 | 0.55 | 2.22 | 0.37 |
| g__*Petrimonas* | 6.11 | 4.07 | 0 | 0 | 0 | - | - | - | - | - | - |
| g__*Thermus* | - | - | - | 0.02 | 0 | 0.03 | 8.15 | 0.09 | 0.13 | 0.31 | 0.29 |
| g__norank_f__TTA-B61 | - | - | 0.27 | 0 | 0.48 | 0.87 | 1.34 | 0.85 | 0.02 | 2.05 | 1.84 |
| g__*Sulfurihydrogenibium* | - | - | - | - | - | - | 8.02 | - | - | - | - |
| g__*Anaerobaculum* | - | - | 1.79 | - | 1.01 | 3.78 | - | - | 0 | - | - |
| g__*Sedimentibacter* | 2.90 | 4.25 | - | - | - | - | - | - | 0 | - | - |
| g__norank_c__Bacteroidetes_vadinHA17 | 2.02 | 3.63 | 0.23 | 0.08 | 0.23 | 0.05 | 0 | 0.02 | 0.04 | - | - |
| g__norank_p__Aminicenantes | 0 | 0 | 0 | - | 0.88 | 4.41 | 0 | - | - | 0 | 0 |
| g__*Longilinea* | 1.76 | 3.52 | 0.14 | 0.03 | 0.11 | 0 | 0 | 0 | 0.02 | - | - |
| g__*Candidatus*_*Caldatribacterium* | 0.08 | 0.17 | 0.19 | 0.10 | 0.50 | 0.61 | 0.57 | 0.68 | 0.04 | 1.69 | 0.21 |
| g__norank_f__Coriobacteriaceae | 0.07 | 1.67 | 0.76 | 0.98 | 0.12 | 0.77 | 0.05 | - | 0.07 | - | 0 |
| g__*Defluviitoga* | - | - | 2.07 | 0.01 | 1.73 | 0.48 | - | - | 0 | 0 | 0 |
| g__norank_c__Candidatus_Nomurabacteria | 2.92 | 2.06 | - | - | - | - | - | - | - | - | - |
| g__*Christensenellaceae*_R-7_group | 1.40 | 2.20 | 0.07 | 0.11 | 0.11 | 0.04 | 0 | - | 0.04 | - | - |
| g__norank_c__SJA-15 | 0.40 | 2.75 | 0.05 | 0.19 | 0.07 | 0.04 | - | 0.03 | 0.22 | - | - |
| g__unclassified_f__Syntrophomonadaceae | - | - | 0.06 | - | 0.75 | 0.01 | - | 0.38 | 0 | 1.74 | 0.42 |
| g__norank_f__Lentimicrobiaceae | 0.02 | 0.01 | 0.11 | - | 1.28 | 1.28 | - | - | - | - | - |
| g__*Hydrogenibacillus* | - | - | 0.08 | 0.34 | 0 | 0.01 | 0.82 | 0.67 | 0.62 | 0.04 | 0.02 |
| g__*Lutispora* | 0.05 | 0.09 | 0.37 | 0.12 | 0.68 | 0.13 | 1.23 | 0 | 0.05 | - | - |
| g__*Thermogutta* | 0.07 | 0.04 | 0.42 | - | 1.38 | 0.36 | 0.35 | - | - | - | - |
| g__*Thermincola* | - | - | 0.01 | - | 0.05 | 2.30 | - | - | - | - | - |
| g__*Desulfovibrio* | 1.02 | 0.99 | 0.24 | 0.03 | 0.22 | 0.03 | - | - | 0.03 | - | - |
| g__*Caldisericum* | 0.04 | 0.04 | 0.83 | - | 1.29 | 0 | - | - | 0 | - | - |

Under relative abundance, “-” indicates that the genus was not detected while “0” indicates the genus was detected but at extremely low levels.
